# Ionization of Volatile Organics and Nonvolatile Biomolecules Directly from a Titanium Slab for Mass Spectrometric Analysis

**DOI:** 10.3390/molecules26226760

**Published:** 2021-11-09

**Authors:** De-Yi Huang, Meng-Jiy Wang, Jih-Jen Wu, Yu-Chie Chen

**Affiliations:** 1 Department of Applied Chemistry, National Yang Ming Chiao Tung University, Hsinchu 300, Taiwan; hhuang1120@gmail.com; 2 Department of Applied Chemistry, National Chiao Tung University, Hsinchu 300, Taiwan; 3 Department of Chemical Engineering, National Taiwan University of Science and Technology, Taipei 106, Taiwan; mjwang@mail.ntust.edu.tw; 4 Department of Chemical Engineering, National Cheng Kung University, Tainan 700, Taiwan; wujj@mail.ncku.edu.tw

**Keywords:** titanium, ESI, APCI, nonpolar, volatile, ambient ionization

## Abstract

Atmospheric pressure chemical ionization (APCI)-mass spectrometry (MS) and electrospray ionization (ESI)-MS can cover the analysis of analytes from low to high polarities. Thus, an ion source that possesses these two ionization functions is useful. Atmospheric surface-assisted ionization (ASAI), which can be used to ionize polar and nonpolar analytes in vapor, liquid, and solid forms, was demonstrated in this study. The ionization of analytes through APCI or ESI was induced from the surface of a metal substrate such as a titanium slab. ASAI is a contactless approach operated at atmospheric pressure. No electric contacts nor any voltages were required to be applied on the metal substrate during ionization. When placing samples with high vapor pressure in condensed phase underneath a titanium slab close to the inlet of the mass spectrometer, analytes can be readily ionized and detected by the mass spectrometer. Furthermore, a sample droplet (~2 μL) containing high-polarity analytes, including polar organics and biomolecules, was ionized using the titanium slab. One titanium slab is sufficient to induce the ionization of analytes occurring in front of a mass spectrometer applied with a high voltage. Moreover, this ionization method can be used to detect high volatile or polar analytes through APCI-like or ESI-like processes, respectively.

## 1. Introduction

Mass spectrometry (MS) has been widely used as an analytical tool to determine molecular weights and structure information for a variety of analytes. Analytes with different polarities require different ionization methods to generate gaseous analyte ions for MS analysis. Atmospheric pressure ionization methods, such as atmospheric pressure chemical ionization (APCI) [1] and electrospray ionization (ESI) [2], are commonly used ionization methods to introduce and ionize analytes at atmospheric pressure. These two ionization methods can be used to cover the analysis of most analytes possessing different polarities in a wide mass range. Conventional APCI is used to ionize analytes based on corona discharge, that is, gas discharge [1]. The atmospheric air around the APCI sharp metal needle applied with a high voltage is initially ionized, followed by the ionization of solvent molecules which can provide protons to ionize analytes. However, the ESI process is initiated when a high voltage is applied to a metal emitter, leading to a charge re-distribution and separation in the eluted droplet for the formation of the Taylor cone. Owing to the Coulombic repulsion exceeding surface tension, a droplet explosion on the apex of the Taylor cone occurs, resulting in the formation of a number of fine droplets in the air. After solvent evaporation, the Coulombic explosion proceeds repeatedly, leading to the formation of finer droplets. Consequently, gas-phase ions are formed for MS analysis after the solvent molecules escape from the fine droplets. Although ionization in ESI requires multiple steps, gaseous analyte ions are generated in a few to a few hundred μs [3,4], depending on the size of the initially formed droplets released from the ESI metal emitter.

Since the beginning of this century, many ambient ionization methods have been explored [5,6,7,8,9,10,11,12,13,14,15,16,17]. Nevertheless, the ionization mechanisms of APCI and ESI are implemented in many ambient ionization methods, probably because most of the analytes possessing different polarities with a wide mass range can be ionized either via APCI or ESI [18,19,20]. Thus, an ionization method possessing dual ionization functions of APCI and ESI is desirable. Efforts have been devoted in developing dual ion sources [21,22]. In general, several accessories, including a high-voltage power supply and small-diameter metal-made ionization needles/emitters, similar to those used in conventional APCI and ESI, are required. The setup of the ionization method can be simplified if these accessories are eliminated.

Several facile ionization methods have been reported [23,24,25,26,27,28,29]. Among them, paper spray has attracted considerable attention owing to its simplicity [23,24]. A piece of filter paper with a sharp end applied with a high voltage can be simply used as the ionization emitter to ionize analytes deposited on the paper [23,24]. In addition, without applying any voltage, tissue paper with a round shape has been demonstrated to be a useful ionization emitter [25]. It has been used as a suitable interface to couple Raman spectroscopy with MS [25]. The thin fibers on the tissue paper presumably played the role of the ionization emitter. Although no voltage was directly applied to the tissue paper, the ionization of analytes occurred due to the polarization of the sample droplet on the tissue paper, induced by the high electric field provided by the mass spectrometer [25,26]. Moreover, contactless atmospheric pressure ionization was reported by using a sharp and tapered capillary of a few centimeters (e.g., 1 cm) as the sampling tube and ESI emitter without having any direct electric contact on the capillary [27]. The short capillary was vertically placed close to the inlet of the mass spectrometer. The capillary inlet was placed into a sample droplet, and the sample was directed to the capillary outlet through the capillary action. Mass spectra with multiple-charge ions of large molecules were obtained. This setup only requires a sharp capillary. In addition, a simplified approach of depositing a microliter-sized sample droplet onto a dielectric substrate without any electric contact was used to ionize the sample when the substrate was placed close to the inlet of the mass spectrometer [28]. Charge separation occurred on the microliter-sized droplet on a dielectric substrate under the influence of an electric field provided by a mass analyzer. As a consequence, the Taylor cone was formed on the sample droplet towards the inlet of the mass spectrometer to initialize ionization of analytes through ESI. The analyte signal was able to last for a few minutes [28]. Multiple-charged ions derived from peptides and proteins were observed. These setups are simple because a high-voltage power supply, metal ionization emitters, and electric cables are eliminated. Nevertheless, the abovementioned ionization methods primarily target analytes with high polarities. In addition, direct analysis in real time (DART) has attracted much attention because of the simplicity and ease-of-operation [6]. A gas-based discharged plasma source is required to initialize the ionization of analytes in different forms. DART can be applied to polar and nonpolar analytes. The main limitation of DART is the upper mass limit. DART is not suitable for the analysis of large proteins. 

Herein, we demonstrate a new ionization approach, that is, atmospheric pressure surface-assisted ionization (ASAI), which can be used not only as an ESI-like source, but also as an APCI-like source. Analytes with different polarities and a wide mass range can be ionized by this ionization method. Only a small metal slab was required for loading the sample and assisting analyte ionization through APCI and ESI. Samples in either the gas phase or condensed phase can be directly ionized at atmospheric pressure by using this approach.

## 2. Experimental

The details of reagents, materials, and instrumentation are provided in the Supporting Information. 

**Figure 1:**
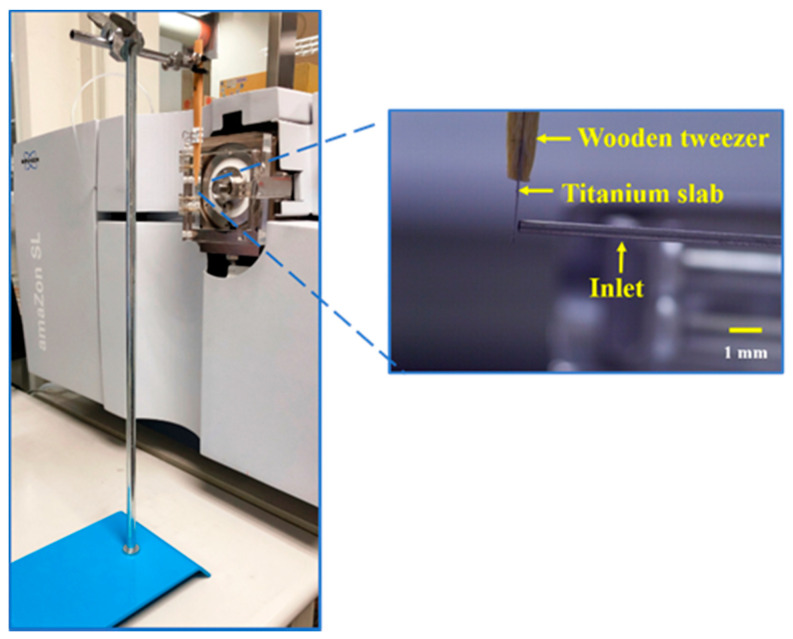
Photograph of the titanium slab-based ASAI–MS setup.

ASAI–MS Analysis. Titanium slabs with different surface areas (0.2 cm × 0.2 cm–4 cm × 4 cm; thickness: ~127 μm) were used as substrates to assist ionization in ASAI–MS. Nevertheless, the titanium slab with XY dimensions of 0.3 cm × 0.3 cm was used as the substrate in most experiments in this work. Figure 1 shows the setup of our ASAI. The surface of the titanium slab held by a pair of wooden tweezers was faced toward the inlet of the mass spectrometer (zoom-in photograph in Figure 1) with a given distance (e.g., ~0.1 mm). The voltages set on the inlet of the mass spectrometer were −4500 V and +4500 V when operating at positive and negative ion modes, respectively. The distance was determined by placing the slab close to the inlet of the mass spectrometer as the ion signals derived from analytes appeared. According to our experience, the ion signal was improved by placing the slab as short a distance from the inlet as possible. However, the slab should not be attached to the inlet of the mass spectrometer. Otherwise, the ion signal disappeared. No electric contacts nor voltages were applied on the titanium slab. When analyzing analytes through APCI, a sample droplet (e.g., 2 µL) was dried on the titanium slab. Once the mass spectrometer was switched on, analyte ions were immediately acquired. Alternatively, a sample containing high-volatility analytes in liquid or solid form was placed underneath the MS inlet within a short distance (e.g., ~1 cm or less). Analyte ions were immediately acquired by the mass spectrometer once the mass spectrometer was switched on. When using the ASAI approach for the analysis of high-polarity analytes, a sample droplet (e.g., 2 µL) containing analytes was deposited on the center of the titanium slab, which was placed close to the inlet of the mass spectrometer (~0.1 mm). The MS running solvent was a mixture of methanol and deionized water (1:1, *v*/*v*). However, the solvent composition was not limited to this solvent mixture. The deionized water was present to maintain the surface tension of the sample droplet to a certain extent. Other organic solvents mixed with deionized water may also be feasible. The choice of solvent was dependent on the solubility of target analytes. When protein samples were analyzed by the ASAI approach, 1% of acetic acid was added to the running solvent. Once the mass spectrometer was switched on, mass spectra were recorded simultaneously.

## 3. Results and Discussion

### 3.1. Examination of Titanium Slab-Based ASAI–MS Analysis through APCI

**Figure 2:**
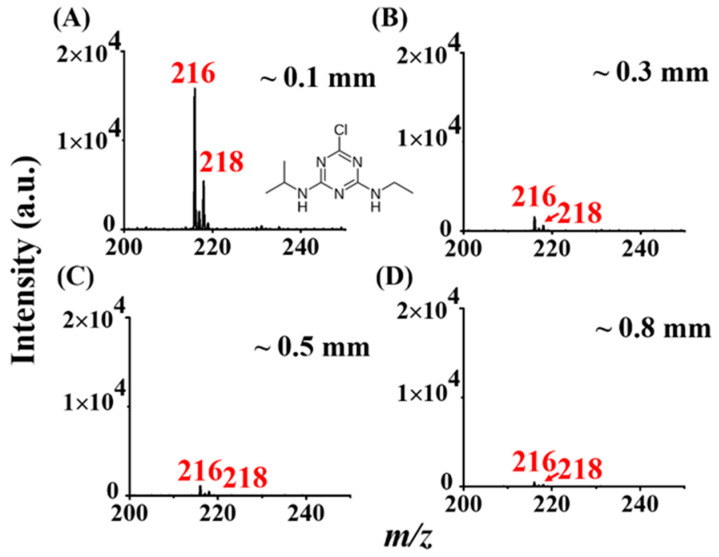
Examination of distance effects. ASAI mass spectra of atrazine were obtained by placing the titanium slab (0.3 cm × 0.3 cm) close to the inlet with a distance of (**A**) ~0.1 mm, (**B**) ~0.3 mm, (**C**) ~0.5 mm, and (**D**) ~0.8 mm. The voltage set on the inlet of the mass spectrometer was −4500 V.

In general, an APCI emitter is made of metal with a sharp tip [1]. A relatively high electric field that is sufficient for inducing corona discharge in ionizing analytes occurs on the sharp metal needle. APCI combined with MS has never been reported with the use of a flat substrate. Nevertheless, point-to-plane setup is not uncommon in generating corona discharge [29]. The use of a flat substrate as the APCI emitter has several advantages, including ease of availability and ease of loading samples. Thus, we initially used a thin titanium slab (0.3 cm × 0.3 cm) as the ionization substrate to examine if the ionization of analytes can occur. Atrazine (vapor pressure: 2.89 × 10^−7^ mmHg at 25 °C [30]) was initially selected as the model sample. The sample droplet containing atrazine (~0.2 nmole) was directly deposited on the center of the titanium slab (0.3 cm × 0.3 cm) (Figure 1). After solvent evaporation, the surface of the slab was placed close to the inlet of the mass spectrometer. The mass spectrum (Figure 2A) derived from the sample was immediately obtained after the mass spectrometer was switched on. The inset in Figure 2A shows the structure of atrazine. The ion peaks at *m/z* 216 and 218 with a ratio of 3/1 derived from the protonated atrazine, which contains one chlorine atom, were observed in the mass spectrum. The results indicated that the titanium slab could be used as an APCI substrate to assist the ionization of analytes. The distance between the titanium slab and the inlet of the mass spectrometer was close (~0.1 mm) when conducting the ASAI–MS analysis. Although the distance was very short, the narrow space between the slab and the inlet of the mass spectrometer had to be maintained. If the slab touched the inlet of the mass spectrometer, the ion signals disappeared. When we moved the slab slightly (i.e., ~0.3 mm [Figure 2B], ~0.5 mm [Figure 2C], and ~0.8 mm [Figure 2D]) from the inlet of the mass spectrometer, the analyte signals at *m/z* 216 and 218 dramatically declined. In addition, the higher the absolute voltage value was set on the inlet of the mass spectrometer, the better the analyte signal was (Supporting Information Figure S1).

In addition, we used titanium slabs with different XY dimensions (0.2 cm × 0.2 cm [Supporting Information Figure S2A], 0.5 cm × 0.5 cm [Supporting Information Figure S2B], 1.0 cm × 1.0 cm [Supporting Information Figure S2C], 1.5 cm × 1.5 cm [Supporting Information Figure S2D], and 4 cm × 4 cm [Supporting Information Figure S2E]) as the APCI substrates to examine the surface area effects. The results showed that the ion signals at *m/z* 216 and 218 derived from the protonated atrazine were observed in the resultant mass spectra using the titanium slabs with different XY dimensions. However, the ion intensity at *m/z* 216 and 218 was apparently lower when using the titanium slab with a larger surface area than those obtained from using the titanium slabs with smaller surface areas as the APCI substrate. The highest signal intensity derived from the analyte ions was obtained from the titanium slab with XY dimensions of 0.2 cm × 0.2 cm, which had a slightly better performance than the titanium slab with the XY dimensions of 0.3 cm × 0.3 cm (*cf.* Figure 2A). The results suggested that the slab with a small surface area could assist ionization to gain high analyte ion peaks. Although the smaller slabs provided better ionization efficiency, the titanium slab with the XY dimension of 0.3 cm × 0.3 cm was selected for ASAI–MS analysis in most of the following experiments because it was easier to fabricate and handle.

### 3.2. Gold-Coated Glass Slide-Based ASAI–MS Analysis

Although the abovementioned experiments were conducted on the basis of the use of a titanium slab as the ionization substrate, other metal substrates such as a gold-coated glass slide could be used alternatively. Supporting Information Figure S3 shows the ASAI mass spectrum of atrazine (~0.2 nmole) obtained by using a gold-coated glass slide (0.3 cm × 0.3 cm) as the ionization substrate. Protonated atrazine at *m/z* 216 and 218 dominated the mass spectrum. The analyte ion intensity was similar to that obtained when a titanium slab (0.3 cm × 0.3 cm) was used as the ASAI ionization substrate (*cf.* Figure 2A). The results indicated that a gold-coated glass slide had similar functions and performance to a titanium slab. The results also implied that other substrates made of metal can be used as the ASAI substrate to assist analyte ionization. Considering the cost and ease-of-fabrication, titanium slabs were used as the ASAI substrate in the following studies.

### 3.3. ASAI–MS Analysis for High-Volatility Analytes through APCI-Like Processes

**Figure 3:**
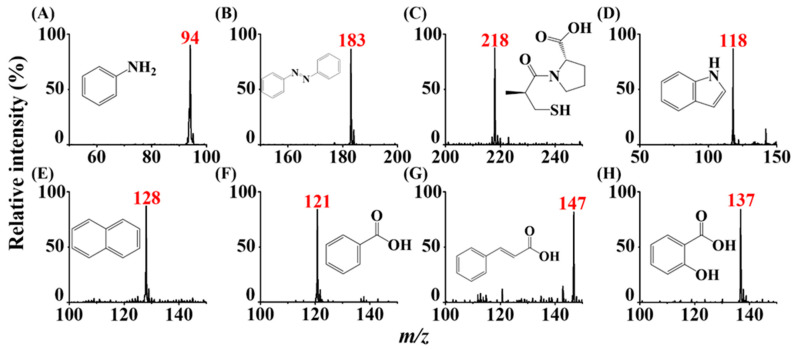
Analysis of analyte vapors derived from liquid samples. ASAI mass spectra of the samples including (**A**) aniline, (**B**) azobenzene, (**C**) captopril, (**D**) indole, (**E**) naphthalene, (**F**) benzoic acid, (**G**) cinnamic acid, and (**H**) salicylic acid prepared in methanol. The concentration of the samples were 10^−4^ M. The vial containing the sample solution (1 mL) was placed under the titanium slab (0.3 cm × 0.3 cm) at a distance of ~1 cm from the surface of the liquid sample. The voltages set on the inlet of the mass spectrometer were −4500 V and +4500 V operated in the positive ion mode and the negative ion mode, respectively.

In addition to directly depositing the sample on the titanium slab, the developed ionization approach was used to detect analyte ion signals from high-volatility samples at atmospheric pressure by placing the samples underneath the titanium slab. The sample was simply placed underneath the inlet of the mass spectrometer at a distance of ~1 cm, and the surface of the titanium slab (0.3 cm × 0.3 cm) was faced toward the inlet of the mass spectrometer at a distance of ~0.1 mm (Supporting Information Figure S4). Aniline (MW = 93 Da), azobenzene (MW = 182 Da), captopril (MW = 217 Da), indole (MW = 117 Da), and naphthalene (MW = 128 Da) were used as the model samples. Supporting Information Table S1 lists their vapor pressures at 25 °C during ASAI–MS analysis. Figure 3A–E shows the resultant mass spectra of the abovementioned analytes, and the insets show the structures of these analytes. Either cationic radicals or protonated molecular ions derived from the analytes dominated the mass spectra, depending on the chemical structures of the analytes. Moreover, this ionization method was used for the detection of negative ions in the negative ion mode. Benzoic acid (MW = 122 Da), cinnamic acid (MW = 148 Da), and salicylic acid (MW = 138 Da) were selected as the model analytes. The vapor pressure of these analytes can be found in Supporting Information Table S1. Deprotonated molecular ions derived from these analytes dominated the resultant mass spectra (Figure 3F–H). These results indicated that our ionization method can be easily used to detect high-volatility analytes either in a positive or negative ion mode with this simple setup. In addition, we examined a variety of compounds which have a high vapor pressure, such as pesticides (Supporting Information Figure S5). Protonated analyte ions dominated the resultant mass spectra. The results showed that our approach can be easily used to detect these samples with a certain vapor pressure at ambient conditions. Moreover, the analyte signal ion disappeared when moving the sample underneath the slab away. Thus, memory effects using this approach were negligible.

### 3.4. Examination of the Lowest Detectable Concentration through the APCI Processe

We examined the lowest detectable concentration of ASAI–MS through the APCI process using atrazine as the model analyte. Supporting Information Figure S6 shows the ASAI mass spectrum of atrazine (10^−7^ M, 2 µL). The signal to noise ratio (S/N) at *m/z* 216 was ~96. The lowest detectable concentration using this approach via the APCI processes was ~10^−7^ M. The intensity of the protonated atrazine was quite apparent, depositing only ~0.4 pmol of the analyte on the titanium slab. 

### 3.5. Ionization Mechanism

Although no electric contact was made directly with the titanium slab, a floating potential was developed because the slab was close (~0.1 mm) to the MS inlet applied with a high voltage. The setup was similar to a point-to-plane corona setup [29]. The metal inlet served as a point electrode, whereas the titanium slab served as a plane electrode. Considering that analyte ions were readily detected by the ASAI–MS approach, gas discharge should occur in the narrow space between the inlet and the slab to initialize ionization of analytes. As mentioned earlier, no analyte ions were obtained if the slab directly touched to the metal inlet of the mass spectrometer. The effects of the distance between the slab and the inlet, the voltage applied to the inlet, and the dimension of the slab on the analyte signal (respectively shown in Figure 2, Figures S1 and S2) suggest that the discharge is developed between the titanium slab and the inlet of the mass spectrometer. Similar phenomena have been previously observed in the point-to-plane corona discharge [29]. 

**Figure 4:**
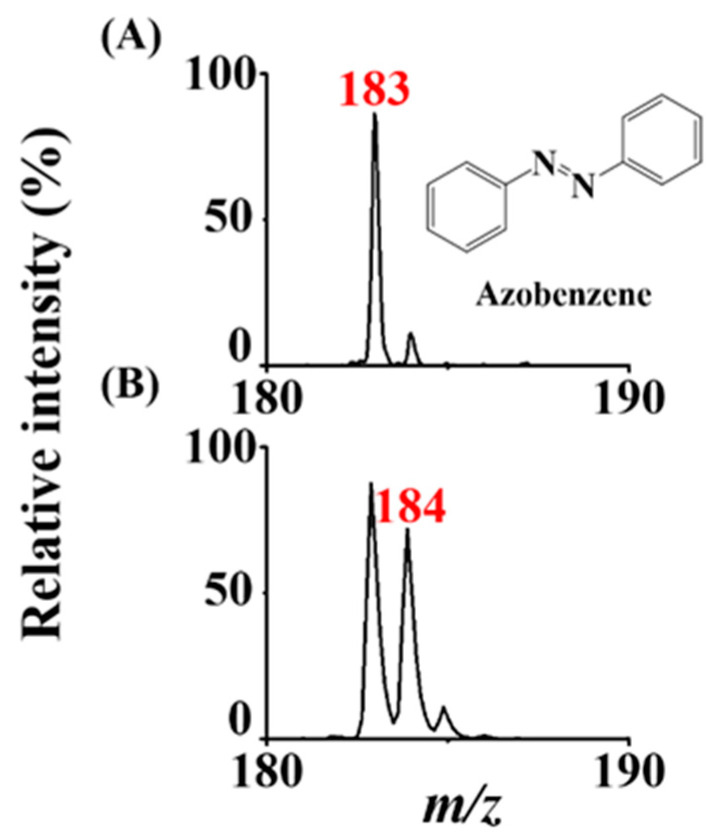
Examination of ionization mechanism. ASAI mass spectra were obtained by depositing azobenzene (0.2 nmole) on the titanium slab (0.3 cm × 0.3 cm) (**A**) without and (**B**) with boiling D_2_O underneath the titanium slab. The distance between the titanium slab and the inlet of the mass spectrometer was ~0.1 mm. The voltage set on the inlet of the mass spectrometer was −4500 V.

In addition, we placed a vial containing boiling D_2_O under the titanium slab deposited with dried azobenzene (~0.2 nmole) to further clarify the ionization mechanism. Figure 4A,B show the ASAI mass spectra of the sample obtained without and with D_2_O underneath the titanium slab, respectively. The ion at *m/z* 183 derived from protonated azobenzene dominated the mass spectrum (Figure 4A), whereas the base ion was shifted to *m/z* 184 (Figure 4B), indicating that D^+^ competed with H^+^ to attach onto the azobenzene. Water molecules from the air are the source of protons during ASAI. The moisture in the laboratory was 40−50%. Trace water molecules in the air are an adequate source of protons for analytes during the ionization process. Presumably, the ionization mechanism is similar to the corona discharge that occurs in conventional APCI, although a metal slab instead of a sharp metal needle was used. Air molecules such as N_2_ and O_2_ were ionized in between the inlet of the mass spectrometer and the titanium slab, followed by ionized water molecules (Reaction 1) in the air, to provide protons for the ionization of gaseous analytes (M) (Reaction 2).

H_2_O^+^·_(g)_+ H_2_O_(g)_ → H_3_O^+^_(g)_ + OH·_(g)_ (1)

H_3_O^+^_(g)_ + M_(g)_ → [M+H]^+^_(g)_ + H_2_O_(g)_ (2)

### 3.6. Direct Analysis of Aroma Molecules from Plants by ASAI–MS Analysis

**Figure 5:**
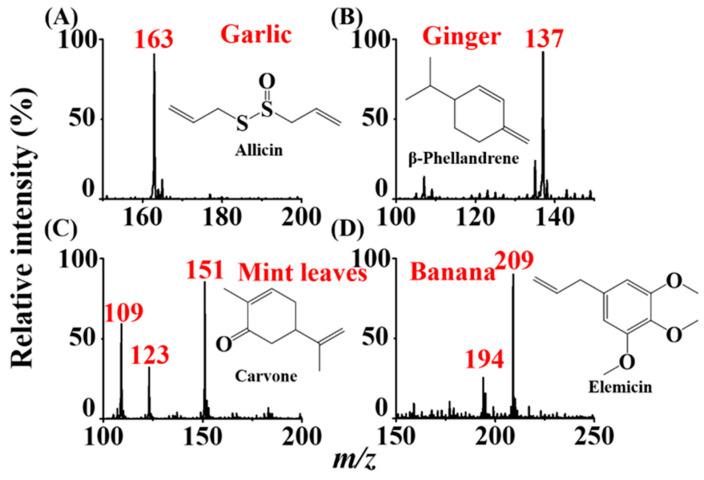
Detection of aroma molecules directly from plants including (**A**) garlic, (**B**) ginger, (**C**) mint leaves, and (**D**) bananas. The samples were placed near the titanium slab (0.3 cm × 0.3 cm) which was placed close to the inlet of the mass spectrometer with a distance of ~0.1 mm. The voltage set on the inlet of the mass spectrometer was −4500 V.

The current approach can be easily used to detect aroma molecules directly from real samples such as garlic, ginger, mint leaves, and bananas. The main aroma molecules immediately appeared in the mass spectra when the sample was placed close to the titanium slab (Supporting Information Video S1). The analyte signal declined quickly when the sample was removed. Figure 5A–D show the resultant mass spectra of garlic, ginger, mint leaves, and bananas, respectively, when placing these sample close to the titanium slab in the ASAI setup. The main volatile species derived from these real samples were allicin (*m/z* 163) [31], β-phellandrene (*m/z* 137) [31], carvone (*m/z* 151) [31] and elemicin (*m/z* 209) [31]. Protonated analyte ions dominated these mass spectra, whereas the fragments derived from these main aroma molecules were also observed in the same mass spectra (Figure 5C,D). The results showed that the main molecules that contributed to an aroma derived from a real sample could be immediately observed by the mass spectrometer. The approach is similar to what has been demonstrated in DART. When the sample was placed close to the DART source in front of the mass spectrometer, the main aroma molecules derived from real samples can be obtained [6]. However, unlike DART, our current approach does not require any additional plasma sources for initializing the ionization of analytes. Only a small titanium slab needs to be used to ionize aroma molecules derived from the real samples. 

### 3.7. ASAI–MS Analysis of Small Organics and Biomolecules through ESI

**Figure 6:**
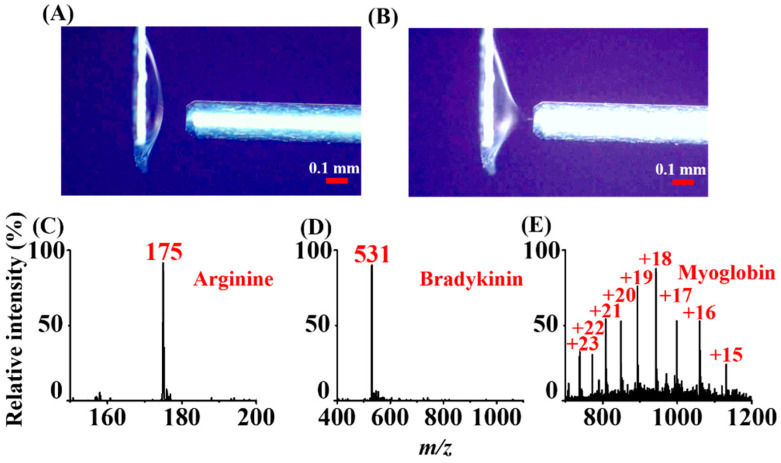
Photographs of a sample droplet deposited on a titanium slab (0.3 cm × 0.3 cm) obtained (**A**) before and (**B**) after applying the high voltage (−4500 V) to the inlet of the mass spectrometer. ASAI mass spectra obtained from individual sample droplets (2 μL) containing (**C**) arginine ([M + H]^+^ = 175), (**D**) bradykinin ([M + 2H]^2+^ = 531), and (**E**) myoglobin (MW = 16,950 Da) deposited on the titanium slab (0.3 × 0.3 cm) followed by ASAI–MS analysis through the ESI-like process.

Moreover, polar analytes can be detected using the current ASAI method through ESI-like processes. After depositing 2 µL of the sample on the titanium slab (0.3 cm × 0.3 cm), a hemisphere droplet was formed (Figure 6A). A Taylor cone immediately appeared (Figure 6B) when the slab loaded with the sample was placed close to the inlet of the mass spectrometer applied with a high voltage. Analyte ions derived from the sample appeared in the mass spectrum immediately (Supporting Information Video S2). Arginine, bradykinin, and myoglobin were selected as model analytes. Protonated arginine ions at *m/z* 175 (Figure 6C), doubly charged bradykinin ions at *m/z* 531 (Figure 6D), and multiple-charged myoglobin ions (Figure 6E) dominated the mass spectra. The results demonstrated that a Taylor cone can be readily formed to initialize ESI for ionizing these biomolecules when depositing a sample droplet onto the titanium slab at a short distance to the inlet of the mass spectrometer (~0.1 mm).

In addition, we examined a variety of analytes, including aflatoxin B1, aflatoxin G1, melamine, ochratoxin A, and ractopamine, which were contaminants or unpleasant residues commonly found in food products. The analysis steps were similar to those obtained in Figure 6. Supporting Information Figure S7 shows the resultant mass spectra. Protonated analyte ions dominated the mass spectra, and the sodium and potassium adducts were also observed in the same mass spectra. These results indicated that the ASAI approach can be used to assist the ionization of analytes with high polarities through the ESI process.

### 3.8. Examination of the Lowest Detectable Concentration and Memory Effects through the ESI-Like Process

We also used a peptide, i.e., DEVDGRRC (DC-8), MW = 949 Da) (10^−6^ M, 2 µL), as the model sample to examine the lowest detectable concentration of this approach through the ESI-like process. Supporting Information Figure S8 shows the resultant ASAI mass spectrum of DC-8, which was dominated by ions at *m/z* 475, 486, and 494 corresponding to [M + 2H]^2+^, [M + H + Na]^2+^, [M + H + K]^2+^, respectively. The S/N at *m/z* 475 was ~32. The lowest detectable concentration of DC-8 using this approach was 10^−6^ M. It is understandable because the Taylor cone formed on the titanium slab was bigger than that eluted from the micro-sized emitter, leading to poorer ionization efficiency. Nevertheless, the Taylor cone can be further reduced by modifying the surface of the ASAI ionization substrate. We are working on this improvement. 

In addition, given that the titanium slab was used as the sample loading substrate, in addition to being the ionization substrate, we also investigated the memory effects. Figure S9A shows the ASAI mass spectrum of the sample containing DC-8 at a relative high concentration, i.e., 10^−5^ M. The doubly charged ions at *m/z* 475 dominated the mass spectrum. After no signal ions were observed in the mass spectra, we deposited the MS running solvent (2 µL) containing methanol and deionized water (1:1, *v/v*) at the same position as the DC-8 sample was deposited and collected the mass spectra. Figure S9B shows the resultant ASAI mass spectrum. A very weak ion at *m/z* 475 was observed. However, the other solvent was deposited at the same position as the solvent was deposited to obtain Figure S9B. No analyte ions were observed (Figure S9C). The results indicated that the sample can be cleaned up by simply being deposited with the MS running solvent. Nevertheless, it is possible that analytes may remain on the substrate if they have a strong interaction with titanium. If it happens, it is also possible to remove the retained species by further rinsing with suitable solvents. 

### 3.9. Application of ASAI–MS to the Analysis of Complex Samples

A complex sample comprising of FBS (fetal bovine serum) spiked with atrazine was used as the simulated sample. FBS is quite complex; thus, direct detection of atrazine in FBS by MS without conducting any sample pretreatment is a challenge. Given that the current approach can ionize analytes with different polarities through either ESI or APCI processes, the analysis of one droplet of a sample from ESI to APCI in a series is possible. A droplet of the FBS sample containing atrazine was deposited on the titanium slab. An ESI mass spectrum was obtained first (Supporting Information Figure S10A). The ion at *m/z* 203, presumably derived from sodium adduct of glucose, dominated the mass spectra. A very weak peak at *m/z* 216, derived from protonated atrazine, was observed in the mass spectrum. After the droplet dried, the ion at *m/z* 203 disappeared, whereas the ion at *m/z* 216 and 218, standing for protonated atrazine, started to appear in the mass spectrum (Supporting Information Figure S10B). The change of the ion signal can be also clearly observed from the extracted ion chromatogram at *m/z* 203 and 216 (Supporting Information Figure S10C). The ion signal at *m/z* 203 lasted for the first 80 s. The ion at *m/z* 216 started to dominate the mass spectrum when the ion at *m/z* 203 dramatically declined, indicating that the sample droplet was dried and the ionization mechanism was dominated by the APCI-like process. These results indicated that our approach is potentially useful for detecting analytes with different polarities through ESI and APCI processes in a series simply from one sample droplet. Moreover, sample pretreatment can be reduced or eliminated. 

## 4. Conclusions

In general, polar and non-polar analytes require different ionization methods for MS analysis. In this study, we have successfully developed a simple ionization approach, ASAI, on the basis of the use of a titanium slab as the ion source. The titanium slab can assist the ionization of analytes with different polarities either through APCI-like or ESI-like processes. Because only a small piece of titanium slab was required for the occurrence of ionization, the ionization method can be easily set up and may be potentially useful for coupling with a portable mass spectrometer. In addition, protein samples can be readily analyzed by ASAI–MS through the ESI process. Although the sensitivity obtained so far from the ESI-like process was not as good as that obtained by conventional ESI-MS, we believe that it can be further improved by modifying the surface of the ASAI substrate to reduce the size of the Taylor cone. Moreover, on-site sample extraction for specific analytes on the ASAI substrate may be possible. For example, phosphorylated species have a high affinity with metal ions such as titanium ions. Thus, on-site extraction of phosphorylated species can be carried out on the slab, and the species trapped on the slab can be analyzed directly by the ASAI–MS approach. Furthermore, the substrate that can be used to assist the ionization of analytes in ASAI–MS analysis is not limited to titanium slabs. We have demonstrated that gold-coated glass slides can also be used. That is, the results suggested that a variety of materials can be used as the substrates to assist ASAI–MS analysis. The main advantages of this approach include simplicity, low cost, simple setup, speed, and a wide detectable mass range with varied polarities. Thus, we are quite optimistic about the further development and applications of this approach in the future.

## Data Availability

The data presented in this study are available in this article and its Supplementary Materials.

## Supplementary Materials

The following are available online, Figure S1: Examination of voltage effects, Figure S2: Examination of the XY dimension effects of the titanium slab, Figure S3: Gold coated substrate-based ASAI–MS, Figure S4: Photograph of the setup of ASAI with a liquid sample underneath the titanium slab, Figure S5: ASAI mass spectra of pesticides, Figure S6: Examination of the lowest detectable concentration of ASAI–MS analysis based on APCI-like processes, Figure S7: ASAI mass spectra of the samples obtained via the ESI-like process, Figure S8: Examination of the lowest detectable concentration in ASAI–MS analysis through the ESI-like process, Figure S9: Examination of memory effects, Figure S10: ASAI–MS analysis results obtained by depositing a 20-fold dilute FBS sample, Table S1: List of the molecular weights and vapor pressure of analytes, Video S1: APCI-based ASAI, Video S2: ESI-based ASAI.
