# Supplementary material for: Ionization of Volatile Organics and Nonvolatile Biomolecules Directly from a Titanium Slab for Mass Spectrometric Analysis
Source: Molecules. 2021 Nov 9;26(22):6760. doi: 10.3390/molecules26226760 (PMC8623480; doi:10.3390/molecules26226760)
Supplement: Supplementary file 1 [file molecules-26-06760-s001.zip › Supplementary materials/Supporting Information_ASAI_Molecules_updated .pdf]

Supporting Information

Ionization of Volatile Organics and Nonvolatile Biomolecules Directly from a Titanium Slab for Mass Spectrometric Analysis

De-Yi Huang,[1,2] Meng-Jiy Wang,[3] Jih-Jen Wu,[4] Yu-Chie Chen[1,2*]

[1]Department of Applied Chemistry, National Yang Ming Chiao Tung University, Hsinchu 300, Taiwan.
[2]Department of Applied Chemistry, National Chiao Tung University, Hsinchu 300, Taiwan
[3]Department of Chemical Engineering, National Taiwan University of Science and Technology, Taipei 106, Taiwan
[4]Department of Chemical Engineering, National Cheng Kung University, Tainan 700, Taiwan
[*]Corresponding author
Email: yuchie@mail.nycu.edu.tw
Tel: +886-3-5131527

Additional Experimental Details

Reagents and Materials

Aniline was purchased from Acros-Organics (Geel, Belgium). p-Cresol were purchased from Alfa Aesar (Ward Hill, MA, USA). Carbofuran, diazinon, melamine, methamidophos, methomyl, parathion, and salicylic acid were purchased from Chem Service (West Chester, PA, USA). Naphthalene was purchased from Fluka (St. Gallen, Switzerland). Acetonitrile, methanol, ethanol, and toluene were obtained from Merck (Darmstadt, Germany), Macron Fine Chemicals (Center Valley, PA, USA), Echo Chemical (Miaoli, Taiwan), and Avantor Performance Materials (Corporate Parkway, PA, USA), respectively. Ametryn, atrazine, benzoic acid, carbazol, chlorpyrifos, malathion, prometon, prometryn, and ractopamine were acquired from Riedel de Haën (Seelze, Germany). Acetic acid, aflatoxin B1, aflatoxin G1, arginine, azobenzene, bradykinin, captopril, D_2O, indole, myoglobin, ochratoxin A, titanium slabs (15 cm × 15 cm; thickness: ~ 0.127 mm), fetal bovine serum, and tetracycline were purchased from Sigma-Aldrich (St. Louis, MO, USA). Fetal bovine serum (FBS) was obtained from Biological Industries (Kibbutz Beit Haemek, Israel). Cover glass slides (1.8 cm × 1.8 cm, thickness: 0.13−0.17 mm) were obtained from Matsunami (Osaka, Japan). Banana, mint leaves, garlic, and ginger were obtained from a local market.

Instrumentation

All the mass spectra were obtained using a Bruker Daltonics AmaZon SL mass spectrometer (Bremen, Germany). When operating at the positive ion mode, −4500 V was set on the orifice of the mass spectrometer. However, +4500 V was set on the orifice of the mass spectrometer when negative ion mass spectra were acquired. The nebulizer was switched-off during the ASAI-MS analysis. The temperature of the ion transfer capillary was set to 200 ^{o}C. The number of the collecting ions set at the ion charge control was 100,000, whereas the maximum acquisition time was set to 100 ms. The length of the metal extension tube (inner diameter: ~1.0 mm; outer diameter: ~1.5 mm) adapted to the orifice of the mass spectrometer was ~4 cm. Gold coated glass slides were fabricated using a metal sputter from Cressington (Watford, UK). The current was set to 30 mA, the sputtering time was set to 35 s. A camera (SG-210X) from Sage Vision (New Taipei City, Taiwan) was used for recording and taking images during MS analysis.

Table S1. List of the molecular weights and vapor pressure of analytes examined by ASAI-MS.

Analyte	MW (monoisotopic)	Vapor pressure* (mm Hg) at 25°C
Ametryn	227.12	2.76×10^{-6}
Atrazine	215.09	2.89×10^{-7}
Prometryn	241.14	1.24×10^{-6}
Prometon	225.16	2.30×10^{-6}
Acephate	183.01	1.70×10^{-6}
Carbofuran	221.10	5.40×10^{-7}
Chlorpyrifos	348.92	1.87×10^{-5}
Malathion	330.03	1.78×10^{-4}
Methamidophos	141.00	3.50×10^{-5}
Methomyl	162.04	5.40×10^{-6}
Diazinon	304.10	9.01×10^{-5}
Aniline	93.06	6.67×10^{-1}
Captopril	217.08	7.25×10^{-6}
Indole	117.06	1.22×10^{-2}
Methylimidazole	82.05	7.00×10^{-3}
Azobenzene	182.08	3.60×10^{-4}
Naphthalene	128.06	7.80×10^{-2}
benzoic acid	122.03	7.00×10^{-4}
Cinnamic acid	48.05	3.21×10^{-5}
Salicylic acid	138.03	8.20×10^{-5}
Endosulfan	403.82	1.73×10^{-7}
Arginine	174.11	-
Bradykinin	1059.56	-
Myoglobin	16950	-
AFB1	312.06	2.26×10^{-10}
AFG1	328.06	2.65×10^{-10}
Melamine	126.07	3.59×10^{-10}
Ochratoxin A	403.08	$3.11 \times 10\text{-}^{14}$
Ractopamine	301.17	6.40×10^{-11}
Tetracycline	444.15	-
2-Phenylphenol	170.07	2.00×10^{-3}

*All the information was obtained from PubChem (https://pubchem.ncbi.nlm.nih.gov)

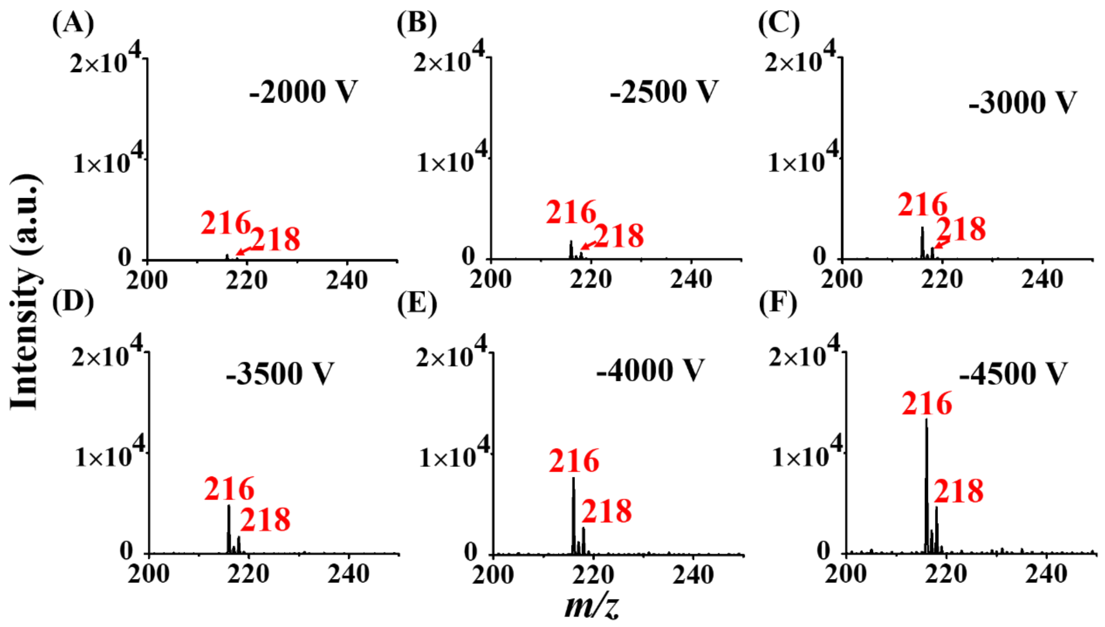

Figure S1. Examination of voltage effects. ASAI mass spectra of atrazine obtained by placing the titanium slab (~0.3 cm × ~0.3 cm) from the inlet with the distance of ~0.1 mm with different voltages applied on the orifice of the mass spectrometer.

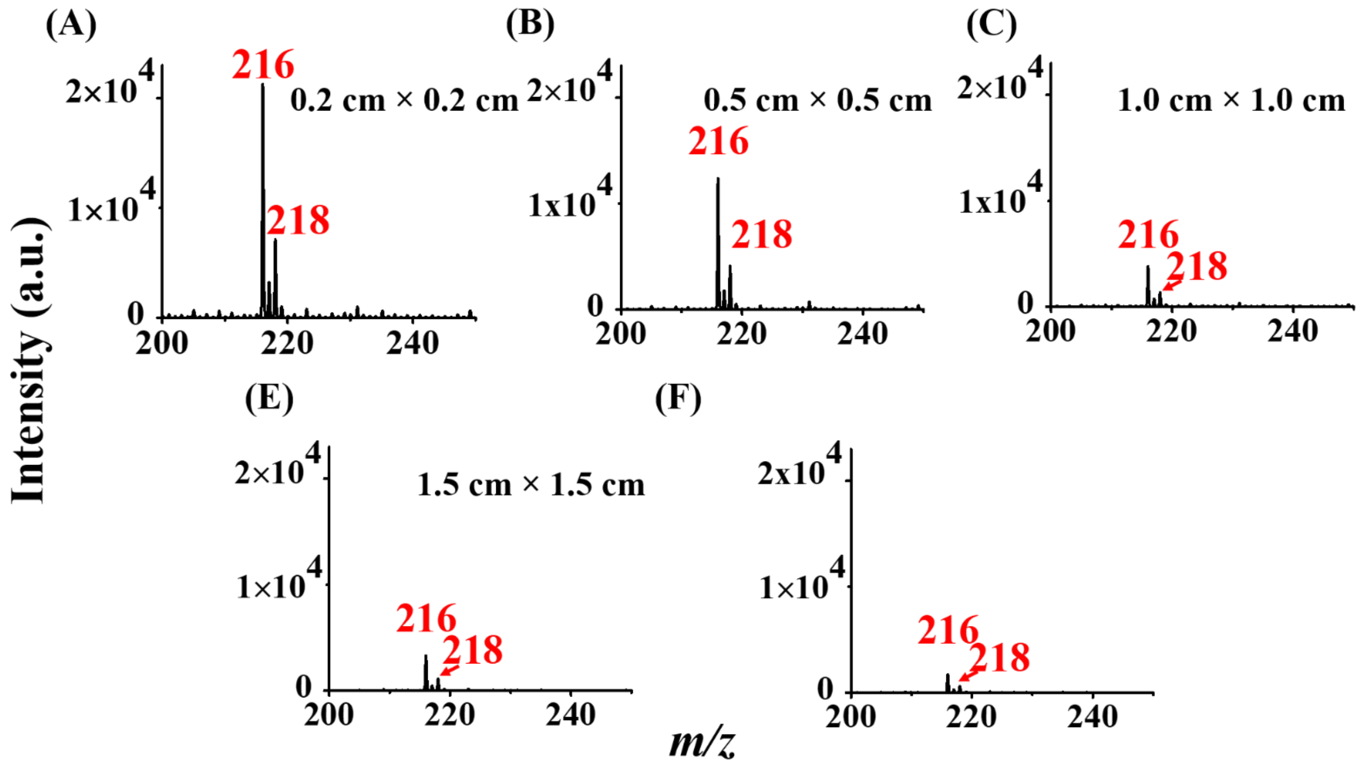

Figure S2. Examination of the XY dimension effects of the titanium slab. ASAI mass spectra of atrazine obtained by placing the titanium slab with the XY dimensions of (A) 0.2 cm × 0.2 cm, (B) 0.5 cm × 0.5 cm, (C) 1.0 cm × 1.0 cm, (D) 1.5 cm × 1.5 cm, and (E) 4.0 cm × 4.0 cm from the inlet with the distance of ~0.1 mm. The voltage of -4500 V was applied on the orifice of the mass spectrometer.

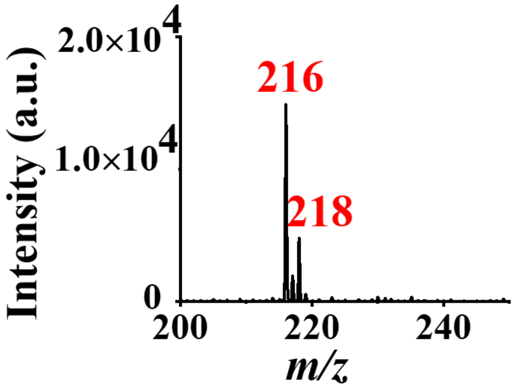

Figure S3. Gold coated substrate-based ASAI-MS. Using a gold coated glass slide (0.3 cm × 0.3 cm) as the ionization substrate. The sample and experimental parameters were the same as those used in Figure 2A.

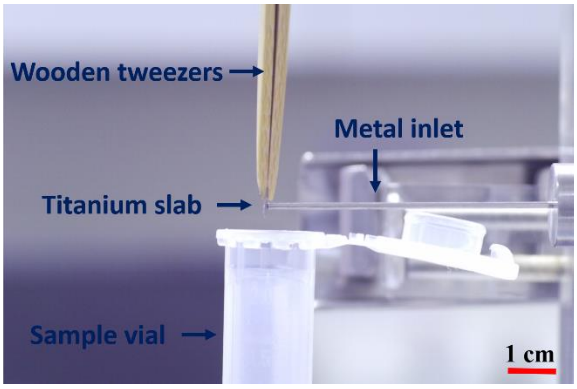

Figure S4. Photograph of the setup of ASAI with a liquid sample underneath the titanium slab.

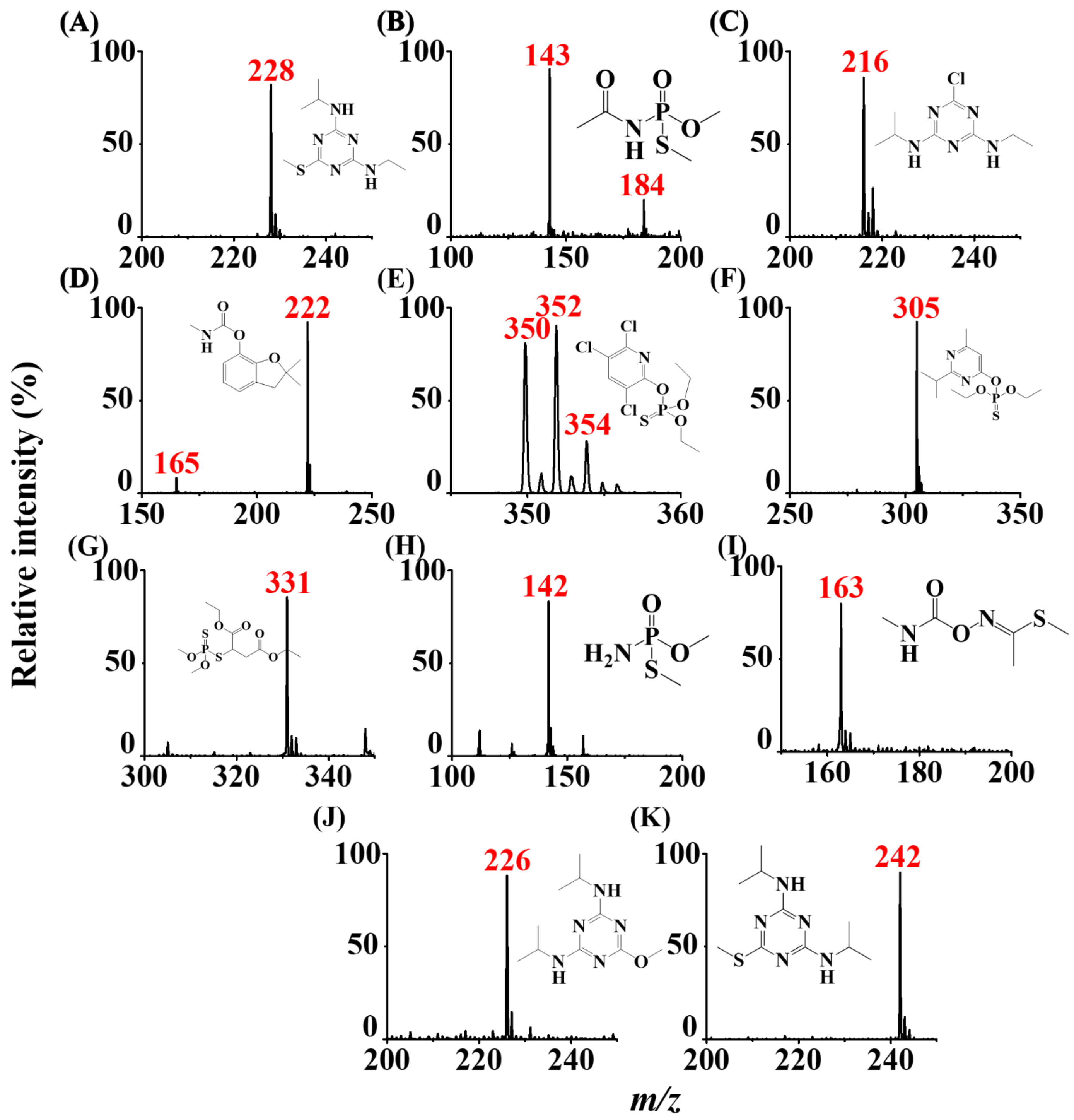

Figure S5. ASAI mass spectra of the samples including (A) ametryn ($[M+H]^+$=228),(B) acephate ($[M+H]^+$= 184), (C) atrazine ($[M+H]^+$= 216), (D) carbofuran ($[M+H]^+$= 222), (E) chlopyrifos ($[M+H]^+$= 350), (F) diazinon ($[M+H]^+$=305), (G) malathion ($[M+H]^+$=331), (H) methamidophos ($[M+H]^+$= 142), (I) methomyl ($[M+H]^+$= 163), (J) prometon ($[M+H]^+$= 226), and (K) prometryn ($[M+H]^+$= 242). The liquid samples were individually placed underneath the titanium slab (0.3 cm × 0.3 cm) for ASAI-MS analysis. The slab was placed from the inlet of the mass spectrometer with the distance of ~0.1 mm during the ASAI-MS analysis. The voltage of −4500 V was applied on the orifice of the mass spectrometer when acquiring ions at the positive ion mode.

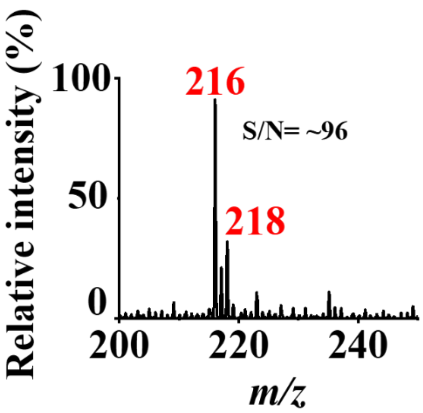

Figure S6. Examination of the lowest detectable concentration of ASAI-MS analysis based on APCI-like processes. ASAI mass spectrum of the sample obtained by depositing atrazine (100 nM, 2 μL) on a titanium slab (0.3 cm × 0.3 cm). After the sample was dried, the slab was placed close (~0.1 mm) to the inlet of the mass spectrometer applied with the voltage of −4500V.

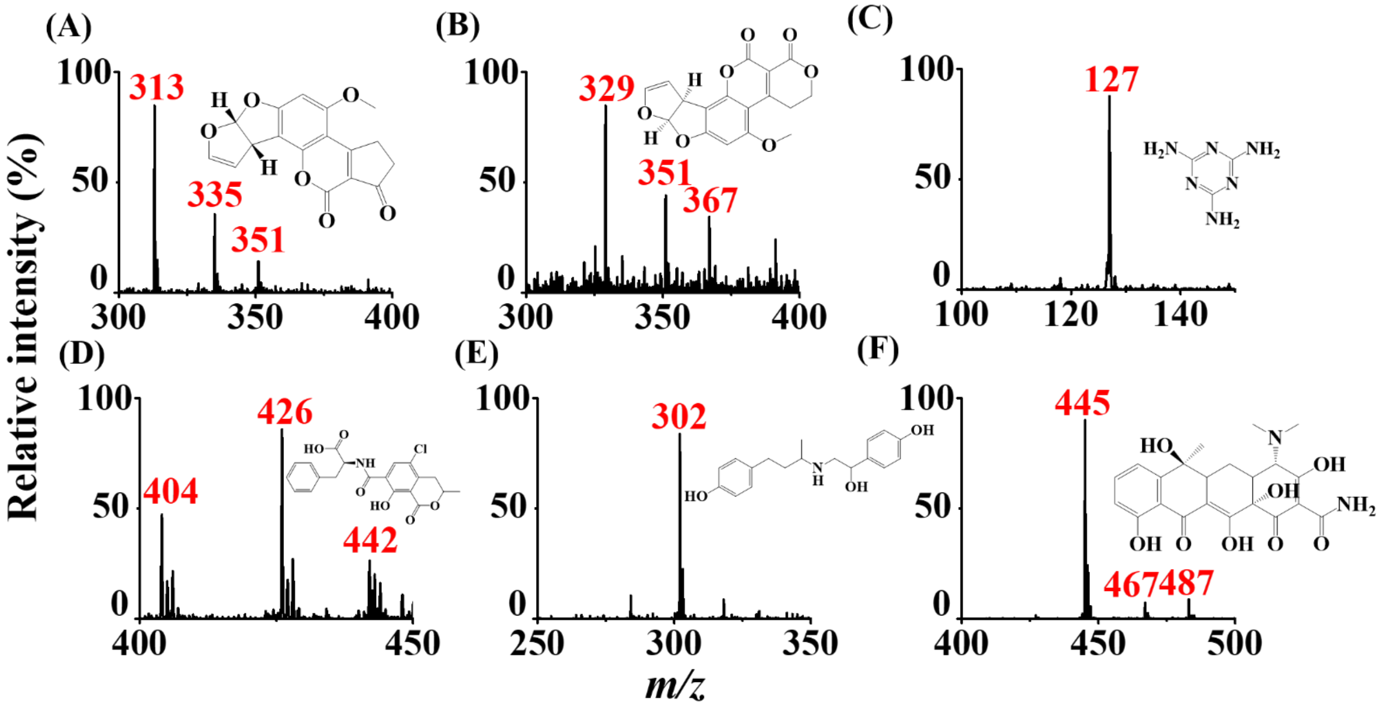

Figure S7. ASAI mass spectra of the samples obtained by depositing iniduival sample droplet (2 uL) containing (A) alfatoxin B1 ([M+H]$^+$= 313), (B) alfatoxin G1 ([M+H]$^+$= 329), (C) melamine ([M-H]$^-$= 127), (D) orchatoxin A ([M+H]$^+$= 404), (E) ractopamine ([M+H]$^+$= 302), and (F) tetracycline ([M+H]$^+$= 445) on a titanium slab (0.3 cm × 0.3 cm) followed by placing the indivdial slab close (~0.1 mm) to the inlet of the mass spectrometer applied with −4500 V at the positive ion mode and +4500 V at the negative ion mode. The concentration of all the sample was 10^{-4} M.

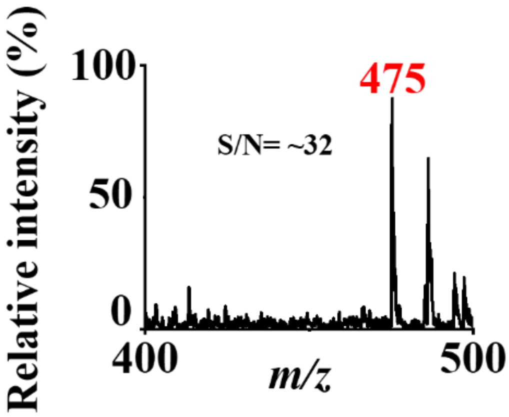

Figure S8. Examination of the lowest detectable concentration in ASAI-MS analysis through the ESI-like processes. ASAI mass spectrum of the sample obtained by depositing a sample droplet (~2 μL) containing DC-8 (10^{-6} M) on a titanium slab (0.3 cm × 0.3 cm) followed by placing the slab close to the inlet of the mass spectrometer applied with the voltage of −4500 V.

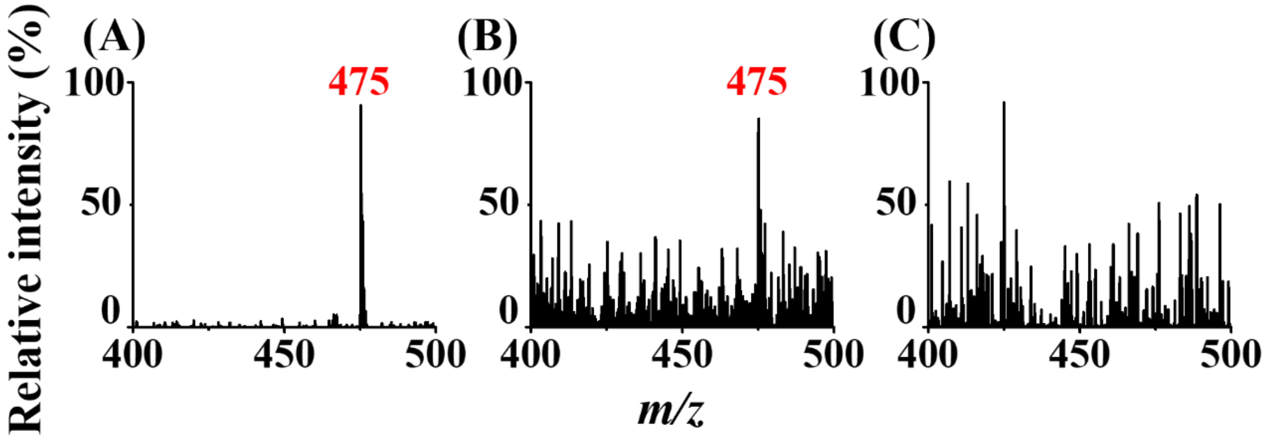

Figure S9. Examination of memory effects in ASAI-MS analysis through the ESI-like processes. (A) ASAI mass spectra of the sample containing DC-8 (10^{-5} M, 2 μL) followed by depositing the MS running solvent (2 μL) for (B) the first and (C) the second time.

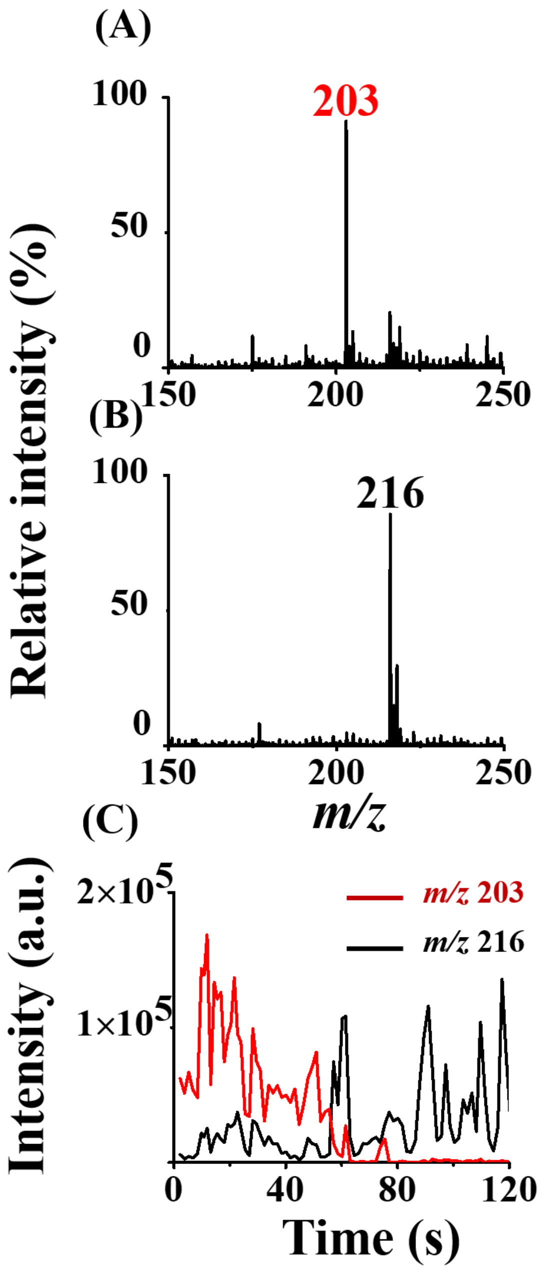

Figure S10. ASAI analysis results obtained by depositing a 20-fold dilute FBS sample droplet containing atrazine (5×10^{-6} M) on a titanium slab (0.3 cm × 0.3 cm) followed by placing the slab close to the inlet of the mass spectrometer with a distance of ~ 0.1 mm. The mass spectra were recorded after the mass spectrometer was switched-on (voltage on the orifice: −4500 V). The mass spectra were continuously monitored for 2 min. ASAI mass spectra of obtained by averaging the mass spectra from the time periods of (A) 0-60 s and (B) 60-120 s. (C) The extracted ion chromatograms at *m/z* 203 (red) and *m/z* 216 (black).
